# Supplementary material for: Ether lipid biosynthesis promotes lifespan extension and enables diverse pro-longevity paradigms in Caenorhabditis elegans
Source: eLife. 2023 Aug 22;12:e82210. doi: 10.7554/eLife.82210 (PMC10444025; doi:10.7554/eLife.82210)
Supplement: MDAR checklist [file elife-82210-mdarchecklist1.docx]

**Materials Design Analysis Reporting (MDAR)**

**Checklist for Authors**

The [MDAR framework](https://osf.io/xfpn4/) establishes a minimum set of requirements in transparent reporting mainly applicable to studies in the life sciences.

*eLife* asks authors to **provide detailed information within their article** to facilitate the interpretation and replication of their work. Authors can also upload supporting materials to comply with relevant reporting guidelines for health-related research (see [EQUATOR Network](http://www.equator-network.org/%20)), life science research (see the [BioSharing Information Resource](http://biosharing.org/)), or animal research (see the [ARRIVE Guidelines](http://www.plosbiology.org/article/info:doi/10.1371/journal.pbio.1000412) and the [STRANGE Framework](https://doi.org/10.1038/d41586-020-01751-5); for details, see *eLife*’s [Journal Policies](https://reviewer.elifesciences.org/author-guide/journal-policies)). Where applicable, authors should refer to any relevant reporting standards materials in this form.

For all that apply, please note **where in the article** the information is provided. Please note that we also collect information about data availability and ethics in the submission form.

**Materials:**

| **Newly created materials** | **Indicate where provided: section/figure legend** | **N/A** |
| --- | --- | --- |
| **Novel *C. elegans* strains**   1. MGH48 mgIs43[ges-1p::GFP::PTS1], RRID: N/A 2. MGH471 (*fard-1* (*oe1*)), RRID: N/A 3. MGH472* (*fard-1* (*oe2*)), RRID: N/A 4. MGH605 (*fard-1* (*oe3*)), RRID: N/A 5. MGH606 (*fard-1* (*oe4*)), RRID: N/A 6. MGH607 (GFP::PTS1; FARD-1::RFP), RRID: N/A | **Novel strains full details and descriptions provided in**  Materials and Methods, *C. elegans* genetics. Available upon request from the Lead Contact. (*MGH472 not available, extra-chromosomal array was lost while attempting long term storage.)  **Novel strains are used within Figure Legends:**  Figure 2—figure supplement 2  Figure 5  Figure 5—figure supplement 1  Figure 6 |  |
|  |  |  |
| **Antibodies** | **Indicate where provided: section/figure legend** | **N/A** |
| N/A | N/A | N/A |
|  |  |  |
| **DNA and RNA sequences** | **Indicate where provided: section/figure legend** | **N/A** |
| **Genotyping primers for:**   1. BX275   F: 5’- tttgctgctgtaggaagagGC - 3’ (wt specific)  R’: 5’- tttgctgctgtaggaagagAC - 3’ (mutant specific)  R: 5’- caattcaccagatgaacagtgg - 3’ (common primer)   1. BX259   F: 5’- tgaagagattttccaacacGA - 3’ (wt specific)  F’: 5’- tgaagagattttccaacacAA - 3’ (mutant specific)  R: 5’- gtctagacgcgtcaaaattcc - 3’ (common primer)   1. BX10   F: 5’- tgaggtttcgaacgactcgCT - 3’ (wt specific)  F’: 5’- tgaggtttcgaacgactcgTT - 3’ (mutant specific)  R: 5’- aagaagtcgatcagcatgagg - 3’ (common primer)  **Quantitative RT-PCR primers for genes:**   1. *act-1* 2. *pmp-3* 3. *fard-1 (spanning exons 5-6 or native 3’ UTR)* 4. *acl-7* 5. *ads-1* | **Genotyping primers provided in** MDAR checklist, DNA and RNA sequences  **Genotyping primers are used within Figure Legends:**  Figure 1  Figure 1—figure supplement 1  Figure 2  Figure 6  Figure 6 – figure supplement 3  **qRT-PCR primers provided in** Materials and Methods, Quantitative RT-PCR  **qRT-PCR primers are used within Figure legend:**  Figure 2—figure supplement 2  Figure 5 |  |
|  |  |  |
| **Cell materials** | **Indicate where provided: section/figure legend** | **N/A** |
| Cell lines: Provide species information, strain. Provide accession number in repository OR supplier name, catalog number, clone number, OR RRID. | N/A | N/A |
| Primary cultures: Provide species, strain, sex of origin, genetic modification status. | N/A | N/A |
|  |  |  |
| **Experimental animals** | **Indicate where provided: section/figure legend** | **N/A** |
| ***C. elegans* strains**   1. N2 (wild type strain), RRID: WB-STRAIN:WBStrain00000001 2. BX275 fard-1(wa28), RRID: WB-STRAIN:WBStrain00004025 3. BX259 acl-7(wa20), RRID: WB-STRAIN: WBStrain00004024 4. BX10 ads-1(wa3), RRID: WB-STRAIN:WBStrain00004007 5. CB1370 daf-2(e1370), RRID: WB-STRAIN: WBStrain00004309 6. MQ989 isp-1(qm150), RRID: WB-STRAIN: WBStrain00026672 7. VC533 raga-1(ok701), RRID: WB-STRAIN: WBStrain00035849 8. DA465 eat-2(da465), RRID:WB-STRAIN: WBStrain00005463 9. SPC168 skn-1(lax188), RRID: WB-STRAIN: WBStrain00034420 10. CF3556 agIs6 [dod-24p::GFP], RRID:WB-STRAIN:WBStrain00004921 11. CL2166 dvIs19 [(pAF15)gst-4p::GFP::NLS], RRID:WB-STRAIN:WBStrain00005102 12. EU31 skn-1(zu135), RRID: WB-STRAIN: WB-STRAIN:WBStrain00007251 13. MGH48 mgIs43[ges-1p::GFP::PTS1], RRID: N/A 14. MGH471 (*fard-1* (*oe1*)), RRID: N/A 15. MGH472* (*fard-1* (*oe2*)), RRID: N/A 16. MGH605 (*fard-1* (*oe3*)), RRID: N/A 17. MGH606 (*fard-1* (*oe4*)), RRID: N/A 18. MGH607 (GFP::PTS1; FARD-1::RFP), RRID: N/A | **Strains provided in**  Materials and Methods, *C. elegans* genetics  #1-12 Available from the *Caenorhabditis Genetics Center* (CGC).  #13-18 Available upon request from the Lead Contact (*, strain lost during cryopreservation).  **Strains are used within**  all Figure Legends (except Figure 7). | N/A |
| Animal observed in or captured from the field: Provide species, sex, and age where possible. | N/A | N/A |
|  |  |  |
| **Plants and microbes** | **Indicate where provided: section/figure legend** | **N/A** |
| Plants: provide species and strain, ecotype and cultivar where relevant, unique accession number if available, and source (including location for collected wild specimens). | N/A | N/A |
| 1. E. coli OP50–1 (CGC), RRID: WB-STRAIN:WBStrain00041971 2. E. coli HT115 (DE3), RRID: WB-STRAIN:WBStrain00041079 | **RRIDs indicated in**   1. Materials and Methods,   *C. elegans* genetics   1. Materials and Methods,   RNA interference (RNAi) assays  **Bacterial strains used as worm food source within *all* Figures and Supplementary Figure Legends (except Figure 7).**   1. Default food source for non-RNAi experiments 2. RNAi experiments are specified within text as gene “knockdowns” or “RNAi” treatment. |  |
|  |  |  |
| **Human research participants** | **Indicate where provided: section/figure legend) or state if these demographics were not collected** | **N/A** |
| If collected and within the bounds of privacy constraints report on age, sex, gender and ethnicity for all study participants. | N/A | N/A |

**Design:**

| **Study protocol** | **Indicate where provided: section/figure legend** | **N/A** |
| --- | --- | --- |
| If the study protocol has been pre-registered, provide DOI. For clinical trials, provide the trial registration number OR cite DOI. | N/A | N/A |
|  |  |  |
| **Laboratory protocol** | **Indicate where provided: section/figure legend** | **N/A** |
| Provide DOI OR other citation details if detailed step-by-step protocols are available. | **Detailed methods are indicated in** Materials and Methods section where published protocols are not available.   We specifically reference our methods paper on GC/MS analysis for detailed step-by-step protocol. (Pino and Soukas, 2020, and Pino et al., 2013). |  |
|  |  |  |
| **Experimental study design (statistics details) *** | | |
| **For in vivo studies: State whether and how the following have been done** | **Indicate where provided: section/figure legend. If it could have been done, but was not, write “not done”** | **N/A** |
| Sample size determination.   1. **Lifespan analysis:**   Yes, n = 50, alpha = 0.05, beta = 0.80, effect size = 20% difference in lifespan based upon log-rank analysis.     1. **GCMS and LC-MS/MS Lipidomics:**   Yes, N = 3, alpha = 0.05, beta = 0.90, effect size = 50%, sigma = 20% based upon pairwise comparison with t-test or ANOVA with multiple hypothesis testing correction. | 1. **Details indicated in**   Materials and Methods,  Longevity assays   1. **Details indicated in**   Materials and Methods,  GC/MS lipidomics and LC/MS-MS lipidomics |  |
| Randomisation | N/A, since experiments are completed with animals sampled at random from a genetically homogeneous population of *C. elegans*, it does not apply to these experiments. | N/A |
| Blinding | **Lifespan Experiments**  Blindly conducted with assigned numbers to different conditions/replicates (indicated in Materials and Methods, Longevity assays).  **GC/MS and LC/MS-MS**  Blindly conducted with coded numbers assigned to different conditions/replicates (indicated in Materials and Methods, GC/MS lipidomics and LC/MS-MS lipidomics). |  |
| Inclusion/exclusion criteria | **Exclusion criteria**  N/A | N/A |
|  |  |  |
| **Sample definition and in-laboratory replication** | **Indicate where provided: section/figure legend** | **N/A** |
| 1. Figure 1B-G; n=3 2. Figure 1H-I; n=3 3. Figure 2A-G; n=3 4. Figure 3A-B; n= 2 5. Figure 3D-G; n=3 6. Figure 4A-C; n=3 7. Figure 5A-B; n=3 8. Figure 5C; n=2 9. Figure 5D; n = 3 10. Figure 5E-G; n=2 11. Figure 5H-I; n=3 12. Figure 6A; n=2 13. Figure 6B-C; n=3 14. Figure 6D-E; n=3 15. Figure 6F; n=3 [N = at least 10 worms per condition per replicate assessed] 16. Figure 1—figure supplement 1A; n=2 17. Figure 1—figure supplement 1B-C; n=3 18. Figure 1—figure supplement 1D-E; n=3 19. Figure 1—figure supplement 2A-F; n=3 20. Figure 2—figure supplement 1; n=3 21. Figure 2—figure supplement 2B; n= 2 22. Figure 2—figure supplement 2C-D; n=2 [N= 20 worms assessed (5 worms per condition; 3 images per worm (upper/mid/lower intestine)) for a total of 60 images (minus 1*)] 23. *1 image lost from phenformin treatment (replicate 2), image file corrupted, unable to open file. 24. Figure 2—figure supplement 2E; n=2 25. Figure 2—figure supplement 2F; n= 2 26. Figure 2—figure supplement 2G-I; n=3 27. Figure 2—figure supplement 2K-L; n=3 28. Figure 2—figure supplement 2M; n=3 29. Figure 4—figure supplement 1A; n=3 30. Figure 4—figure supplement 1B; n=3 31. Figure 4—figure supplement 1C; n=2 32. Figure 5—figure supplement 1A; n=2 33. Figure 5—figure supplement 1B; n=3 34. Figure 5—figure supplement 1C; n=3 35. Figure 5—figure supplement 1D-F; n=2 36. Figure 6—figure supplement 1A-B; n=2 [N = at least 10 worms per condition per replicate assessed.] 37. Figure 6—figure supplement 2A-G; n=3 38. Figure 6—figure supplement 3A-F; n=2 | **Replicates provided in**  Results and Figure Legends (except for Figure 7).  **Replicates for individual lifespan experiments** are also noted in Supplementary file 1. |  |
| n= independent biological replicates, as a result of independently prepared egg preps, RNAi clones (where applicable), and drug treatment. Additionally, each biological replicate was prepared at a separate time, while samples within the same replicate were prepared simultaneously. | **Replicates provided in**  Figure Legends (except for Figure 7). | N/A |
|  |  |  |
| **Ethics** | **Indicate where provided: section/submission form** | **N/A** |
| Studies involving human participants: State details of authority granting ethics approval (IRB or equivalent committee(s), provide reference number for approval. | N/A | N/A |
| Studies involving experimental animals: State details of authority granting ethics approval (IRB or equivalent committee(s), provide reference number for approval. | N/A | N/A |
| Studies involving specimen and field samples: State if relevant permits obtained, provide details of authority approving study; if none were required, explain why. | N/A | N/A |
|  |  |  |
| **Dual Use Research of Concern (DURC)** | **Indicate where provided: section/submission form** | **N/A** |
| If study is subject to dual use research of concern regulations, state the authority granting approval and reference number for the regulatory approval. | N/A | N/A |

**Analysis:**

| **Attrition** | **Indicate where provided: section/figure legend** | **N/A** |
| --- | --- | --- |
| N/A | N/A | N/A |
|  |  |  |
| **Statistics** | **Indicate where provided: section/figure legend** | **N/A** |
| 1. **Lifespan experiments** are analyzed by log-rank test using online OASIS2. Justification: This a commonly used method to compare survival data between samples. 2. **Determination of body size for *C. elegans*** is performed by two-way ANOVA. Justification: Compared 2 independent variables (drug and RNAi treatment). 3. **Colocalization analysis of FARD-1::RFP and PTS1::GFP** is performed by calculating the Pearson correlation coefficient for the overlap of intestinal RFP and GFP expression in images taken of vehicle or phenformin treated MGH607 worms. Pearson’s r values for images corresponding to 2 replicates of vehicle or drug treated worms were then combined to generate 4 average r values (1 per condition). These averages were compared via an unpaired t-test, 95% CI: [-0.2196, 0.2477]. For vehicle treated worms (N=30, M= 0.611, Mdn= 0.6, SD=0.136, SEM= 0.025). For phenformin treated worms (N=30, M= 0.625, Mdn= 0.64, SD=0.128, SEM= 0.024). Justification: The amount of subjective overlap between RFP and GFP (by eye) corresponded with changes in Pearson’s r values, thereby giving us a means to quantify colocalization. For t-test, we compared the means of 2 groups (vehicle vs. drug treatment.) 4. **Number of lipid droplets present in *glo-4* RNAi vehicle or phenformin treated worms** are analyzed by unpaired t-test, 95% CI: [-56.63, -5.887]. For vehicle treated worms (N= 57, M= 154.474, Mdn= 149, SD= 84.117, SEM=11.142). For phenformin treated worms (N= 90, M= 123.222, Mdn= 114, SD= 70.136, SEM= 7.393). Justification: For t-test, we compared the means of 2 groups (vehicle vs. drug treatment.) 5. **Mean fluorescence intensity measurements for *dod-24p::GFP* and *gst-4p::GFP::NLS* animals treated with vehicle or phenformin on OP50-1 NGM or RNAi treatment plates** were quantified using FIJI/ImageJ, and analyzed using two-way ANOVA followed by Šídák's multiple comparisons testing for pairwise comparisons of drug treatment responses. Justification: Comparing two independent variables followed by pairwise multiple comparisons (drug and RNAi treatment, Figure 6F, Figure 6—figure supplement 1A-C). 6. **GC/MS experiments** are analyzed by unpaired students t-test, multiple t-test, or two-way ANOVA. Justification: For unpaired student’s t-test, we compared the means of 2 groups (wt vs. *fard-1* mutants, Figure 2A-B, drug treatment for each bacterial viability status, Figure 6—figure supplement 2D-F). For two-way ANOVA, we compared conditions with 2 independent variables (strain and drug treatment, Figure 2C-D, bacterial viability status and drug treatment, Figure 6—figure supplement 2C and 2G). For multiple t-test (with two-stage linear step-up procedure of Benjamini, Krieger and Yekutieli): 7. we compared the means of 2 groups (vehicle vs. drug treatment) for more than one metabolite (Figure 2E-G and Figure 2—figure supplement 1) 8. we compared the means of 2 groups (wt vs. *fard-1 (oe3)*) for more than one metabolite (Figure 5G-H) 9. **LC-MS/MS metabolomics** **dataset**: 10. **Comparison of normalized concentrations of biguanide in  *C. elegans*:** two-tailed students t-test with Bonferroni   correction for multiple hypothesis testing. Justification: preceding ANOVA established significant variation in the data, and t-test with post-hoc correction is appropriate to control for multiple hypothesis testing because multiple comparisons (multiple metabolites) were conducted).   1. **Heat map**: one-way ANOVA followed by false discovery rate (FDR) control using the Benjamini-Hochberg (BH) method. Post-hoc testing was then performed using Fisher’s LSD to evaluate pairwise comparison significance. Justification: As for 1 above, ANOVA established significant variation attributable to experimental conditions; thereafter individual comparisons made with t-test corrected for multiple hypothesis testing. 2. **qRT-PCR experiments** are analyzed by unpaired student’s t-test or two-way ANOVA followed by Šídák's multiple comparisons testing. Justification: For unpaired student’s t-test, we compared the means of 2 groups (vehicle vs. phenformin treatment, Figure 2 – figure supplement 2G-L) for each gene. For two-way ANOVA, we compared conditions with two independent variables followed by pairwise multiple comparisons (strain and drug treatment, Figure 5D). | 1. **Analyses for lifespan experiments are provided in**   Materials and Methods, Longevity assays.  **Relevant figure statistics are found in:**  Figure 1  Supplementary File 1  Figure 3  Figure 4  Figure 5  Figure 1—figure supplement 1  Figure 4—figure supplement 1  Figure 5—figure supplement 1  Figure 6—figure supplement 3   1. **Analyses for *C. elegans* body size provided in**   Materials and Methods, Body Size Determination of *C. elegans*  **Relevant figure statistics are found in:**  Figure 1—figure supplement 1   1. **Colocalization statistics detailed in**   Materials and Methods, Colocalization analysis of FARD-1::RFP and peroxisomal targeted GFP  **Relevant figure statistics are found in:**  Figure 2—figure supplement 2     1. **Lipid droplet analysis provided in**   Materials and Methods, Lipid droplet analysis  **Relevant figure statistics are found in:**  Figure 6   1. **Fluorescence intensity analysis for dod-24p::GFP and gst-4p::GFP::NLS animals provided in**   Materials and Methods, Reporter fluorescence quantification analysis.  **Relevant figure statistics are found in:**  Figure 6  Figure 6—figure supplement 1   1. **Analysis for GC/MS provided in**   Materials and Methods, GC/MS lipidomics and Quantification and statistical analysis.  **Relevant figure statistics are found in:**  Figure 2: t-test, two-way ANOVA, multiple t-test  Figure 6—figure supplement 2: t-test, two-way ANOVA.  Figure 5: multiple t-test   1. **Detailed analysis for LC/MS-MS metabolomics provided in**   Materials and Methods, Statistical analysis of metabolomics data.  **LC-MS/MS metabolomics are provided within Figure Legends:**  Figure 1: Comparison of normalized concentrations of biguanide  Figure 2: Heat map   1. **qRT-PCR analysis provided in**   Materials and Methods, Quantitative RT-PCR.  **Analyses for qRT-PCR experiments are provided within Figure Legend:**  Figure 2—figure supplement 2  Figure 5 |  |
|  |  |  |
| **Data availability** | **Indicate where provided: section/submission form** | **N/A** |
| For newly created and reused datasets, the manuscript includes a data availability statement that provides details for access (or notes restrictions on access). | **All GC/MS data are included and analyzed** in their entirety in the main manuscript, Figure 2, Figure 2-figure supplement 1, Figure 5I, and Figure 6 - figure supplement 2.  **All LC/MS data for ether lipid abundances are analyzed** in the main manuscript in Figure 2, and raw, normalized, and normalized/log-transformed data are included in Figure 2-source data 1, as indicated in the main manuscript. Data have also been uploaded to Dryad and can be found at [https://datadryad.org/stash/share/tZw0MURwnUaWP6Y6maavIpvz0tQIvJhRSjhapMSmcmY](https://secure-web.cisco.com/1Cti-hkkYPzp7PdLkowvXBOhOIoELwuoBy2GHfTcW27tzG5uzwkBjEjzkW6m7OTE4L4LWClq3cJ9nURDF--fhqp6oNWmKkgmIP1GTSeh7ZnWPsro4y01Jf1Sh_zGxJj5K3khTl7XnJqwjChWkOk9ARwSiTlEh3g0T4ujMLjoHInQXV7X_tbtcbG-6VI5ISKGG-sUcQMPiDxXergGLGb1avKtm7V1G2Gwh-7x762x5DK88nhqoH0EQTbfKK_RsqcMKqAB0NikxIUTl9wdMtRgBFhLwVND4PX9INoqt7Tey4kq3JmzFOPD8RT0MxrIkrY_f/https%3A%2F%2Fdatadryad.org%2Fstash%2Fshare%2FtZw0MURwnUaWP6Y6maavIpvz0tQIvJhRSjhapMSmcmY)  **Longevity summary data tables are included** for primary datasets and for biological replicates in Supplementary file 1, and this is reflected in each main and supplementary figure legend, where appropriate. |  |
| When newly created datasets are publicly available, provide accession number in repository OR DOI and licensing details where available. | N/A | N/A |
| If reused data is publicly available provide accession number in repository OR DOI, OR URL, OR citation. | N/A | N/A |
|  |  |  |
| **Code availability** | **Indicate where provided: section/figure legend** | **N/A** |
| For any computer code/software/mathematical algorithms essential for replicating the main findings of the study, whether newly generated or re-used, the manuscript includes a data availability statement that provides details for access or notes restrictions. | **CellProfiler parameters used for** analysis of lipid droplets are indicated in:  Materials and Methods, Lipid droplet analysis  **Parameters for MetaboAnalyst analysis of LC/MS-MS data are** **indicated** in the Materials and Methods, Statistical analysis of metabolomics data |  |
| Where newly generated code is publicly available, provide accession number in repository, OR DOI OR URL and licensing details where available. State any restrictions on code availability or accessibility. | N/A | N/A |
| If reused code is publicly available provide accession number in repository OR DOI OR URL, OR citation. | N/A | N/A |

**Reporting:**

The MDAR framework recommends adoption of discipline-specific guidelines, established and endorsed through community initiatives.

| **Adherence to community standards** | **Indicate where provided: section/figure legend** | **N/A** |
| --- | --- | --- |
| State if relevant guidelines (e.g., ICMJE, MIBBI, ARRIVE, STRANGE) have been followed, and whether a checklist (e.g., CONSORT, PRISMA, ARRIVE) is provided with the manuscript. | We adhere to ICMJE standards as  indicated in author contributions section, using the CRediT taxonomy designations.  A PRISMA checklist is not included as it is redundant with this MDAR form, but we also adhere to those standards. |  |
